# Genotypic Variation in a Breeding Population of Yellow Sweet Clover (*Melilotus officinalis*)

**DOI:** 10.3389/fpls.2016.00972

**Published:** 2016-07-12

**Authors:** Kai Luo, M. Z. Z. Jahufer, Fan Wu, Hongyan Di, Daiyu Zhang, Xuanchen Meng, Jiyu Zhang, Yanrong Wang

**Affiliations:** ^1^State Key Laborotary of Grassland Agro-Ecosystems, College of Pastoral Agriculture Science and Technology, Lanzhou UniversityLanzhou, China; ^2^AgResearch Limited, Grasslands Research CentrePalmerston North, New Zealand

**Keywords:** forage breeding, genotypic variation, genotype-by-environment interactions, correlation coefficient, coumarin

## Abstract

Yellow sweet clover is a widely spread legume species that has potential to be used as a forage crop in Western China. However, limited information is available on the genetic variation for herbage yield, key morphological traits, and coumarin content. In this study, 40 half sib (HS) families of *M. officinalis* were evaluated for genotypic variation and phenotypic and genotypic correlation for the traits: LS (leaf to stem ratio), SV (spring vigor), LA (leaf area), PH (plant height), DW (herbage dry weight), SD (stem diameter), SN (stem number), Cou (coumarin content), SY (seed yield), across two locations, Yuzhong and Linze, in Western China. There was significant (*P* < 0.05) genotypic variation among the HS families for all traits. There was also significant (*P* < 0.05) genotype-by-environment interaction for the traits DW, PH, SD, SN, and SV. The estimates of HS family mean repeatability across two locations ranged from 0.32 for SN to 0.89 for LA. Pattern analysis generated four HS family groups where group 3 consisted of families with above average expression for DW and below average expression for Cou. The breeding population developed by polycrossing the selected HS families within group 3 will provide a significant breeding pool for *M. officinalis* cultivar development in China.

## Introduction

Yellow sweet clover known as field melilot or yellow melilot, is an annual or biennial herb that belongs to the Fabaceae family. It is native to temperate and tropical Asia, and Europe (GRIN, 2000). *Melilotus officinalis* is one of the most common species in the *Melilotus* genus. This species has adaptation to environmental constraints such as drought and cold (Turkington et al., 1978) and salinity (Sherif, 2009). *Melilotus* is used as a ground cover in depleted soils (Allen and Allen, 1981), especially in moderately saline areas where traditional forage legumes cannot be grown (Maddaloni, 1986). *Melilotus officinalis* usually occurs in the northern region of China, where it is used as green manure for soil fertility improvement and also as a medicinal plant.

Species of *Melilotus*, including yellow sweet clover, have not been widely used in forage production due to their high coumarin content. Coumarin, a secondary plant metabolite, is associated with dicoumarol production. Dicoumarol is an anticoagulant that can cause a haemorrhagic condition known as sweet clover disease (Evans and Kearney, 2003; Nair et al., 2010). Therefore, the success of forage cultivar development based on any of the *Melilotus* species will depend on a combination of increasing dry matter production and decreasing coumarin content. A number of cultivars of *Melilotus* have been released to date; Acuma, Cumino, Denta, Polara (Smith and Gorz, 1965; Goplen, 1971) for *M. albus* and Norgold (Goplen, 1981), N28, N29 (Gorz et al., 1992) for *M. officinalis*. The *Melilotus* breeding program at Lanzhou University is specifically focused on the development of new cultivars with adaptation to the vast temperate grazing environments of China (Luo et al., 2014).

In any plant breeding program, the rate of genetic gain depends on the genetic diversity for a given trait in the breeding population (Hallauer and Miranda, 1981). Information on the magnitude of genetic variation for key plant attributes in breeding programs will enhance the development of appropriate breeding strategies to achieve maximum genetic gain (Moll and Stuber, 1974). Jahufer and Casler (2015) evaluated the relative merit in genetic gain using single trait selection, correlated response to selection and index selection, based on estimated genetic variation for a range of morphological and quality traits in switch grass (*Panicum virgatum* L.). Genetic variation for key traits have been reported for some of the important forage grasses and legumes: ryegrass (Breese and Hayward, 1972), tall fescue (Piano et al., 2007), white clover (Jahufer et al., 2002), alfalfa (Riday and Brummer, 2007).

There is a lack of quantitative genetic information for *Melilotus*. Few studies have been carried out on the genetic variation for agronomic traits in *Melilotus* species (Ivanov and Chetvertnykh, 1980; Sagalbekov, 1980). Nair et al. (2010) reported genotypic variation for coumarin content among 149 accessions of 15 *Melilotus* species. This study demonstrated the presence of potential genetic variation for coumarin content in *Melilotus* germplasm useful for breeding. However, breeding *Melilotus* species as a forage crop needs to focus on not only coumarin content but also biomass and associated traits. There is also a lack of information on the magnitude of genotype-by-environment interaction effects in *Melilotus*, which will be important for breeding for broad adaptation (Cooper et al., 1993b).

The objective of our study was to conduct a preliminary assessment of the performance of half sib (HS) families of *Melilotus officinalis* across two contrasting locations to: (a) estimate genotypic variation for key traits, and (b) identify families with a combination of superior agronomic performance and low coumarin expression in comparison to two commercial controls.

## Materials and methods

### Plant material

Six germplasm accessions (PI 552553 and PI 552554, PI 595394, PI 634019, Ames 22891, and Ames 25658) were selected from a set of 51 accessions that were evaluated for biomass production, agronomy and low coumarin, in Yuzhong, Gansu Province, during 2012–2013 (results not presented). Elite genotypes representing each of the germplasm accessions were polycrossed in isolation, using honey bees, to generate a breeding population to be used for cultivar development. A total of 40 HS families were generated by harvesting each of the genotypes individually. All harvested seeds from the individual genotypes were kept separately as individual HS families.

### Field trials

**Figure 1:**
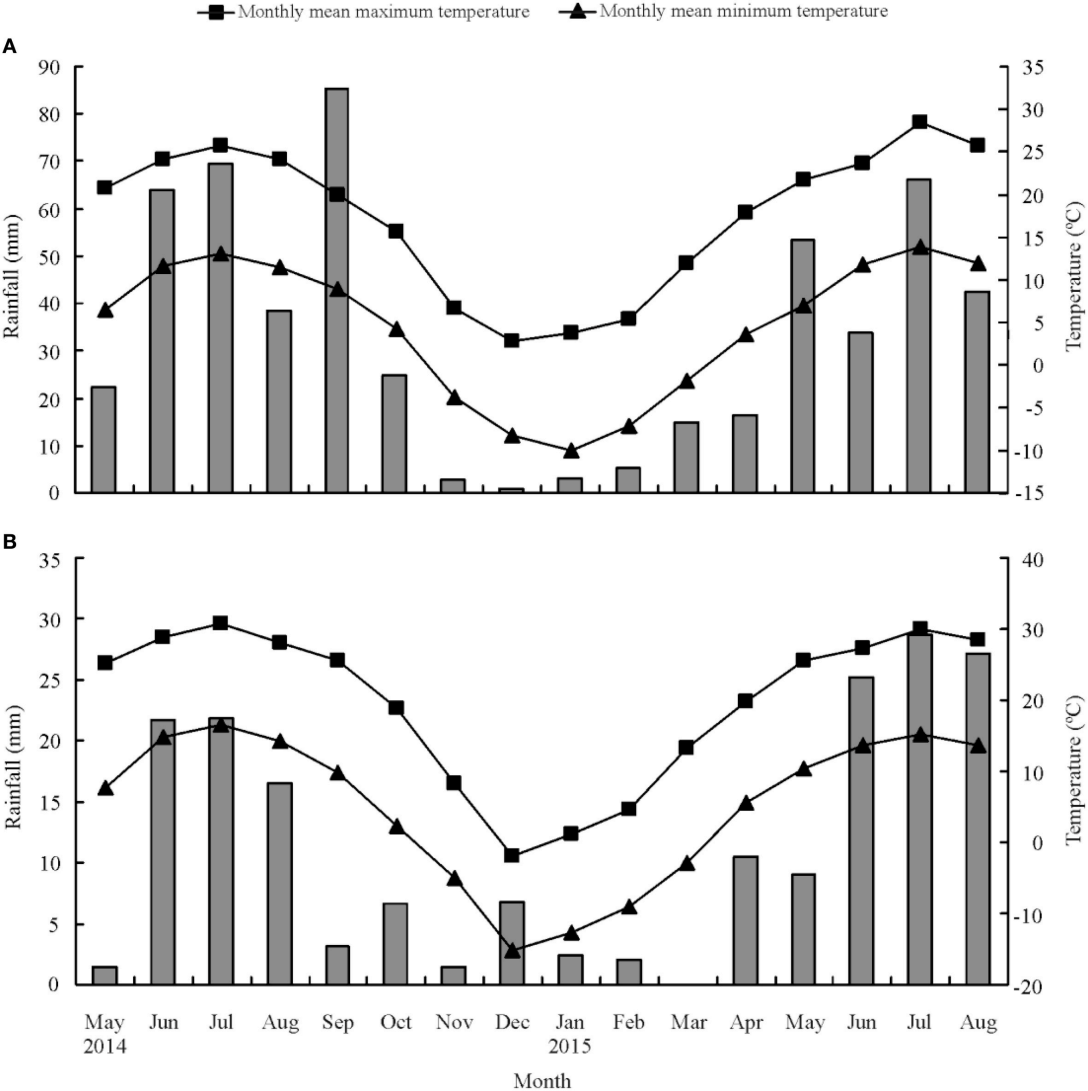
**Mean monthly maximum and minimum temperatures (°C) and total monthly rainfall (mm) at Yuzhong (A) and Linze (B), respectively**.

The *M. officinalis* HS families were established at two locations: Yuzhong (104°09′ E, 35°89′ N, elevation 1 653 m a.s.l.) and Linze (100°02′ E, 39°15′ N, elevation 1 390 m a.s.l.) in Gansu Province, China. There are different climate conditions between Yuzhong and linze. Yuzhong in Loess Plateau region is a medium temperate semi-arid climate, whereas Linze in the Hexi Corridor is typical desert climate and characterized by an arid climate (Su et al., 2007; Hu et al., 2012; Li et al., 2014). The average annual precipitation in Yuzhong is 295 mm and in Linze is only 117 mm. The mean monthly minimum and maximum temperatures, and total monthly rainfall during the trial period at the two locations are shown in Figure 1.

The soil type at each location is loessal soil at Yuzhong and meadow soil at Linze. The saline-alkali degree was much higher in Linze than in Yuzhong, the salinity is 1.8 ppt in Linze and 0.5 ppt in Yuzhong. Initial soil conditions in Yuzhong and Linze are: pH 7.0 and 7.5, total N of 0.756 g/kg and 0.803 g/kg, total P of 0.752 and 0.708 g/kg, respectively.

At each location, the experimental plots were arranged in a randomized complete block design containing three replicates. Each replicate consisted of the 40 HS families the six parental germplasm accessions and two commercial checks. The origins of these entries are provided in Table 1. The two trials were sown in 15–18 June 2014. The experimental plot size for each entry was 2.4 m^2^ (0.8 × 3 m). Within each plot, the seed was planted at a spacing of 30 cm within-rows and 60 cm between-rows. The plots were fertilized with 150 kg (NH_4_)_2_HPO_4_ ha^−1^ after sowing.

**Table 1:** **Origin of the ***M. officinalis*** germplasm accessions and commercial check cultivars**.

**Accession number**	**Source of seed**	**Latitude and Longitude**
PI 552553	Nebraska, United States	41°29′N, 99°54′W
PI 552554	Nebraska, United States	Unknown
Ames 22891	Russia	44°28′N, 40°48′E
Ames 25658	Tien Shan Mountains, China	43°46′N, 89°27′E
PI 595394	Canada	56°07′N, 106°20′W
PI 634019	Saskatchewan, Canada	52°56′N, 106°27′W
Norgold	Nebraska, United States	Commercial cultivar
LX 03	Qinghai, China	Experimental cultivar

### Measurements

The traits measured were: LS, leaf to stem ratio; SV, spring vigor; LA, leaf area (cm^2^); PH, plant height (cm); DW, herbage dry weight (g/plant); SD, stem diameter (cm); SN, stem number; Cou, coumarin (% of dry matter); SY, seed yield (g/plant). All the traits were measured in the second year (2015).

Visual scoring for SV was based on a scale of 1 to 5 (1 = low; 5 = high). The morphological traits (PH, SN, SD, and LA) were measured at the flowering stage (50% of the plants had open flowers), resulting in a minimum of three individuals per replicate. LA was measured from three middle leaflets per plant by using a flatbed scanner (EPSON GT-15000) and a WinSEEDLE 2011 image analysis system (Regent Instruments Inc.). Individual plant was harvested for DW measurement at the flowering stage after measuring morphological traits. At harvest, three randomly sampled plants from each replicate were cut off at 3 cm above the soil, placed in paper bags and dried at room temperature (about 20–25°C) with good ventilation until no change in weight was recorded. After measuring DW, the dried samples were hand separated into leaf blade and stem (including the inflorescence and leaf sheath) components and weighted to determine the LS ratio. Three sub samples from each field replicate at Yuzhong were combined and ground in a mill to pass through a 1 mm screen for Cou determination. SY was determined from two randomly sampled individuals taken from each replicate when 90% of the pods turned blackish brown at the Linze field. Cou was quantified using HPLC (high performance liquid chromatography, Agilent 1100 series) with a mobile phase of methanol-water (65:35) through an Agilent-XDB C18 column (Zhu and Fan, 2008).

### Analysis of variance

The data were analyzed within and across the two locations Yuzhong and Linze. The analysis across locations was conducted: (a) on only the 40 HS families to estimate genotypic variation, and (b) using all entries in the trial that consisted of the 40 HS families, the six parental germplasm accessions and two check cultivars, which enabled comparison of progeny, parents, and the commercial material. The analysis was conducted using the variance component analysis procedure, Residual Maximum Likelihood (REML) option, in GenStat 7.1 (2003). A mixed linear model was used for the analyses across the two locations using the REML algorithm.

The linear model used in the analysis was,

(1) $$\begin{array}{r} Y_{ijk}=M+g_{i}+l_{j}+r_{jk}+{\left(gl\right)}_{ij}+\varepsilon_{ijk}, \end{array}$$

Where, *Y*_*ijk*_ is the value of an attribute measured from HS family *i* in replicate *k* in location *j*, and *I* = 1,…,n_*g*_, *j* = 1,…,n_*l*_, *k* = 1,…,n_*r*_; *M* is the overall mean; *g*_*i*_ is the random genotypic effect of HS family *i*, N(0,σ^2^_*g*_); *l*_*j*_ is the fixed effect of location *j*, N(0,σ^2^_*l*_); *r*_*jk*_ is the random effect of replicate *k* within location *j*, N(0,σ^2^_*b*_); (*gl*)_*i*__*j*_ is the effect between HS family *i* and environment *j*, N(0,σ^2^_*gl*_); ε_*ijk*_ is the residual effect for HS family *i* in replicate *k* in location *j*, N(0,σ^2^_ε_).

The mixed model analysis generated HS family means based on Best Linear Unbiased Predictors (BLUP) (White and Hodge, 1989). These BLUP values were used to construct a HS family × trait mean matrix adjusted of HS family × location interaction effects.

### Genotypic variation and repeatability

Variation among HS families generated from a population that has gone through at least two cycles of random mating, is an estimate of ¼ additive variation of the random mating population they represent (Falconer, 1989). In our study, the 40 HS families were a result of the first random mating of selected germplasm and therefore represented only the F1 generation. Therefore, we do not refer to the variation estimated among the 40 HS families as ¼additive variation, but as genotypic variation, due to a possible combination of additive and non-additive effects. The genotypic variation for the different traits enabled calculation of repeatability, an estimation of the upper limits of their degrees of genetic determination (Falconer, 1989).

The genotypic variance components generated from the REML analysis within and across locations were used to calculate repeatability (*R*) (Fehr, 1987).

HS family mean repeatability at a single site:

(2) $$\begin{array}{r} R_{1}=\frac{\sigma_{g}^{2}}{\sigma_{g}^{2}+\frac{\sigma_{\varepsilon }^{2}}{n_{r}}} \end{array}$$

HS family mean repeatability across locations:

(3) $$\begin{array}{r} R_{2}=\frac{\sigma_{g}^{2}}{\sigma_{g}^{2}+\frac{\sigma_{gl}^{2}}{n_{l}}+\frac{\sigma_{\varepsilon }^{2}}{n_{l}n_{r}}} \end{array}$$

Where, in both model (2) and model (3), the respective variance components and their divisors are defined in relation to linear model (1).

### Phenotypic and genotypic correlation

Phenotypic correlation (*r*_*p*_) analysis was carried out using GenStat 7.1 (2003). The multivariate MANOVA procedure, within GenStat 7.1 (2003), enabled estimation sums of cross-products, using the multisite trait data from the 40 HS families. Mean cross products were then calculated and resolved to estimate genotypic covariance components. The genotypic covariance components were used together with the σ^2^_*g*_ estimates, from REML analysis, to determine genotypic correlation coefficients (*r*_*g*_) according to Falconer (1989).

### Pattern analysis

Pattern analysis was conducted to: (a) provide a graphical summary of the performance of the 40 HS families, six parental germplasm accessions and the two check cultivars of *M. officinalis*, based on the genotype × trait BLUP adjusted mean matrix generated from variance component analysis across the two locations Yuzhong and Linze, and (b) investigate any changes in type (positive or negative) and magnitude of the association among the seven traits across Yuzhong and Linze. Pattern analysis consisted of a combination of cluster and principal component analysis (PCA) (Gabriel, 1971; Kroonenberg, 1994; Watson et al., 1995). To identify the optimum level of truncation for the resulting hierarchy from cluster analysis, the increase in the sum of squares among accession groups was monitored as the number of groups increased. The group level selected was determined by the point where the percentage of accession sum of squares among groups did not improve substantially as the number of groups increased (DeLacy, 1981).

## Results

### Genotypic variance components and HS family mean repeatability of plant attributes of *M. officinalis*

The genotypic variance estimated for the different traits from the individual location, Yuzhong and Linze, analysis indicated significant (*P* < 0.05) variation among the 40 *M. officinalis* HS families (Tables 2A,B). At both these locations HS family mean repeatability estimates ranged from intermediate to very high, depending on the traits.

**Table 2A:** **Average, maximum, minimum, least significant differences (***l.s.d***._**0.05**_), genotypic ($\sigma_{g}^{2}$), and experimental error ($\sigma_{\varepsilon }^{2}$) variance components and associated standard errors (±SE), and HS family mean repeatability (***R***_**1**_) estimated from the 40 ***M. officinalis*** half sib families, evaluated at Yuzhong**.

**Traits**	**DW**	**LS**	**PH**	**SD**	**SN**	**SV**	**LA**	**Cou**
Average	128	0.93	163	1.83	8.2	2.2	7.6	0.44
Max	216	1.22	192	2.77	11.4	4.7	8.3	1.14
Min	69	0.63	136	1.22	7.0	0.8	6.8	0.14
*l.s.d._0.05_*	18	0.17	12	0.28	1.38	0.779	0.54	0.133
$\sigma_{g}^{2}$	1709 ± 393	0.025 ± 0.006	236 ± 57	0.193 ± 0.046	1.213 ± 0.336	0.890 ± 0.218	0.24 ± 0.06	0.093 ± 0.021
$\sigma_{\varepsilon }^{2}$	232 ± 17	0.032 ± 0.002	153 ± 11	0.069 ± 0.005	2.43 ± 0.18	0.208 ± 0.030	0.114 ± 0.017	0.004 ± 0.001
*R*_1_	0.96	0.70	0.82	0.89	0.60	0.93	0.86	0.86

*LS, leaf to stem ratio; SR, spring vigor; LA, leaf area (cm^2^); PH, plant height (cm); DW, herbage dry weight (g/plant); SD, stem diameter (cm); SN, stem number; Cou, coumarin (% of dry matter)*.

**Table 2B:** **Average, maximum, minimum, least significant differences (***l.s.d***._0.05_), genotypic ($\sigma_{g}^{2}$), and experimental error ($\sigma_{\varepsilon }^{2}$) variance components and associated standard errors (±SE), and HS family mean repeatability (***R***_**1**_) estimated from the 40 ***M. officinalis*** half sib families, evaluated at Linze**.

**Traits**	**DW**	**LS**	**PH**	**SD**	**SN**	**SV**	**LA**	**SY**
Average	125	0.96	168	1.55	8.4	2.0	7.8	7.1
Max	207	1.18	190	2.63	11.5	3.5	8.5	10.3
Min	45	0.77	139	0.66	7.5	0.1	6.8	4.7
*l.s.d._0.05_*	19	0.17	13	0.24	1.61	0.47	0.62	1.74
$\sigma_{g}^{2}$	1792 ± 413	0.015 ± 0.004	190 ± 47.3	0.233 ± 0.054	1.18 ± 0.36	1.32 ± 0.31	0.24 ± 0.06	2.19 ± 0.59
$\sigma_{\varepsilon }^{2}$	264 ± 19	0.040 ± 0.003	170 ± 12	0.047 ± 0.003	3.80 ± 0.27	0.11 ± 0.02	0.08 ± 0.01	2.48 ± 0.23
*R*_1_	0.95	0.53	0.46	0.94	0.77	0.97	0.90	0.73

*LS, leaf to stem ratio; SV, spring vigor; LA, leaf area (cm^2^); PH, plant height (cm); DW, herbage dry weight (g/plant); SD, stem diameter (cm); SN, stem number; SY, seed yield (g/plant)*.

At Yuzhong, the HS family mean repeatability (*R*_1_) was high for the traits DW, SD and SV, which ranged from 0.89 to 0.96 (Table 2A). For the traits PH, LA and Cou, HS family mean repeatability was high (0.82–0.86). HS family mean repeatability was intermediate (0.60 and 0.70) for SN and LS. At Linze, HS family mean repeatability was very high (0.90–0.97) for the traits LA, SD, DW, and SV (Table 2B). The traits SY and SN had high (0.73, 0.77) HS family mean repeatability. HS family mean repeatability was intermediate (0.46, 0.53) for PH and LS.

Analysis of variance for mean trait expression across the two sites Yuzhong and Linze indicated significant (*P*<*0.05*) genotypic variation among the 40 HS families. There was also significant (*P* < 0.05) genotype-by-location interaction, depending on the traits (Table 3). There was no significant (*P* > 0.05) genotype-by-location interaction for the traits LS and LA. Line mean repeatability (*R*_2_) across the two locations varied from: relatively high for the traits DW and LA; intermediate for PH, LS, SD, and SV; and low for SN.

**Table 3:** **Average, maximum, minimum, least significant differences (***l.s.d***._**0.05**_), genotypic ($\sigma_{g}^{2}$), genotype-by-location interaction ($\sigma_{gl}^{2}$), and experimental error ($\sigma_{\varepsilon }^{2}$) variance components and associated standard errors (±SE), and HS family mean repeatability (***R***_**2**_) estimated from the 40 ***M. officinalis*** half sib families, evaluated across two locations, Yuzhong and Linze**.

**Traits**	**DW**	**LS**	**PH**	**SD**	**SN**	**LA**	**SV**
Average	126	0.94	165	1.69	8.3	7.7	2.1
Max	206	1.20	188	2.57	11.6	8.4	3.8
Min	63	0.71	144	1.72	7.4	6.7	0.5
*l.s.d._0.05_*	14	0.13	9	0.2	1.16	0.38	0.55
$\sigma_{g}^{2}$	1025 ± 327	0.018 ± 0.005	102 ± 40	0.099 ± 0.038	0.504 ± 0.257	0.24 ± 0.06	0.56 ± 0.21
$\sigma_{gl}^{2}$	715 ± 167	ns	109 ± 28	0.112 ± 0.027	0.637 ± 0.227	ns	0.53 ± 0.13
$\sigma_{\varepsilon }^{2}$	279 ± 14	0.036 ± 0.002	167 ± 9	0.065 ± 0.003	3.52 ± 0.18	0.09 ± 0.01	0.18 ± 0.02
*R*_2_	0.72	0.60	0.55	0.60	0.36	0.89	0.65

*LS, leaf to stem ratio; SV, spring vigor; LA, leaf area (cm^2^); PH, plant height (cm); DW, herbage dry weight (g/plant); SD, stem diameter (cm); SN, stem number*.

*ns, not significant (p <0.05)*.

### Pattern analysis: Principal component analysis (PCA)

**Figure 2:**
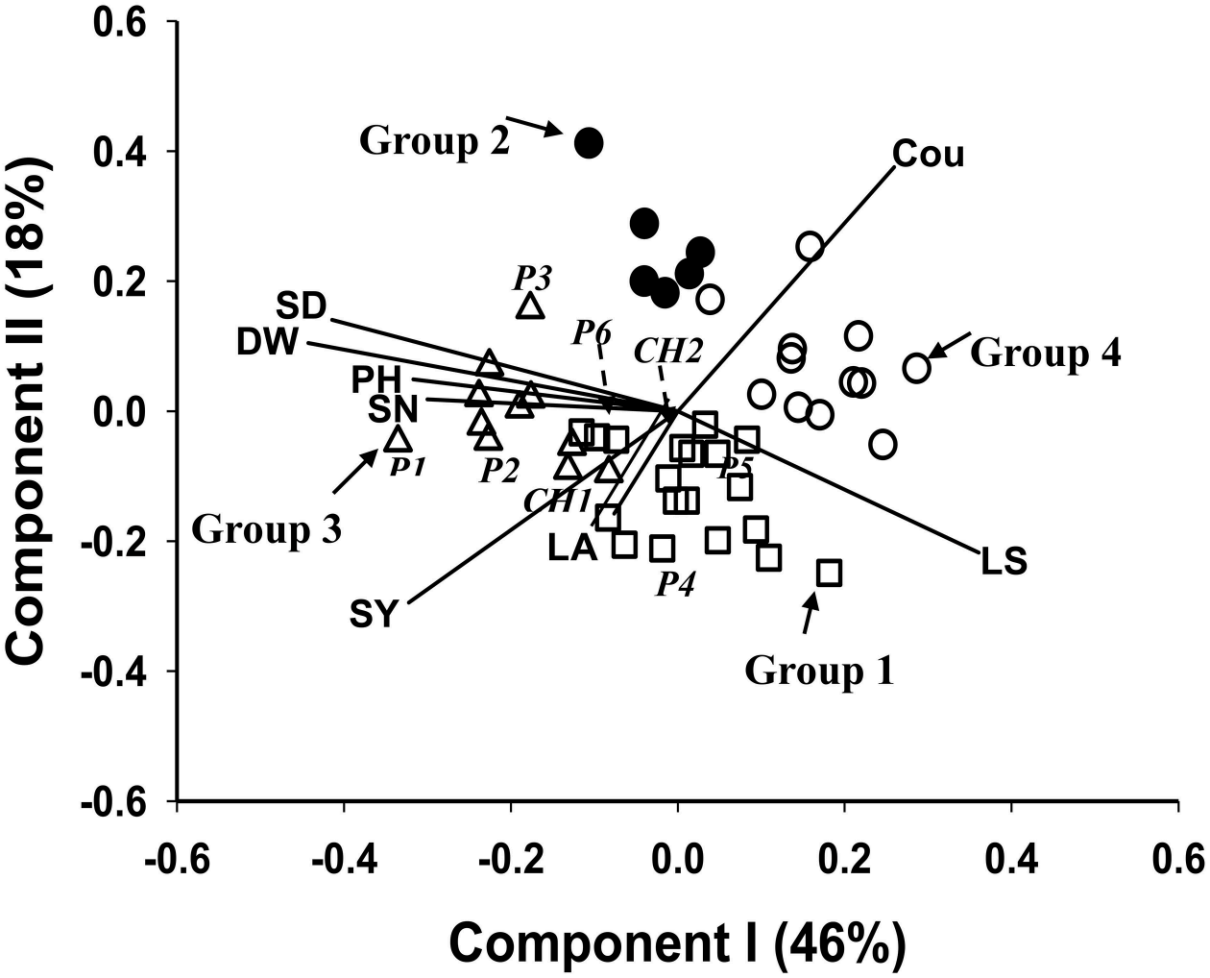
**Biplot generated using standardized Best Linear Unbiased Predictor values for eight traits measured from: the 40 half sib families, the 6 parental germplasm accessions and the 2 check cultivars of ***M. officinalis***, evaluated across two locations Yuzhong and Linze**. Components I and II account for 46 and 18% of total variation, respectively. The different symbols indicate progeny Groups 1 to 4 generated from cluster analysis. The vectors represent the traits: LS, leaf to stem ratio; LA, leaf area; PH, plant height; DW, herbage dry weight; SD, stem diameter; SN, stem number; SY, seed yield; Cou, coumarin. The 6 parental germplasm accessions: *P*1 to *P*6. Check's: CH1, experimental cultivar; CH2, cv Norgold.

The biplot (Figure 2) was generated from PCA of the 40 HS families, the six parental germplasm accessions and the two check cultivars of *M. officinalis*, based on the 9 traits LS, SV, LA, PH, DW, SD, SN, and Cou. The first principle component explained 46% of the total trait variation, and the second principle component explained 18%. The correlation structure of the traits is indicated by the directional vectors in the biplot. In this study, SD, SN, and PH showed a strong positive association with DW. The traits LS and Cou also showed a negative correlation with DW.

**Figure 3:**
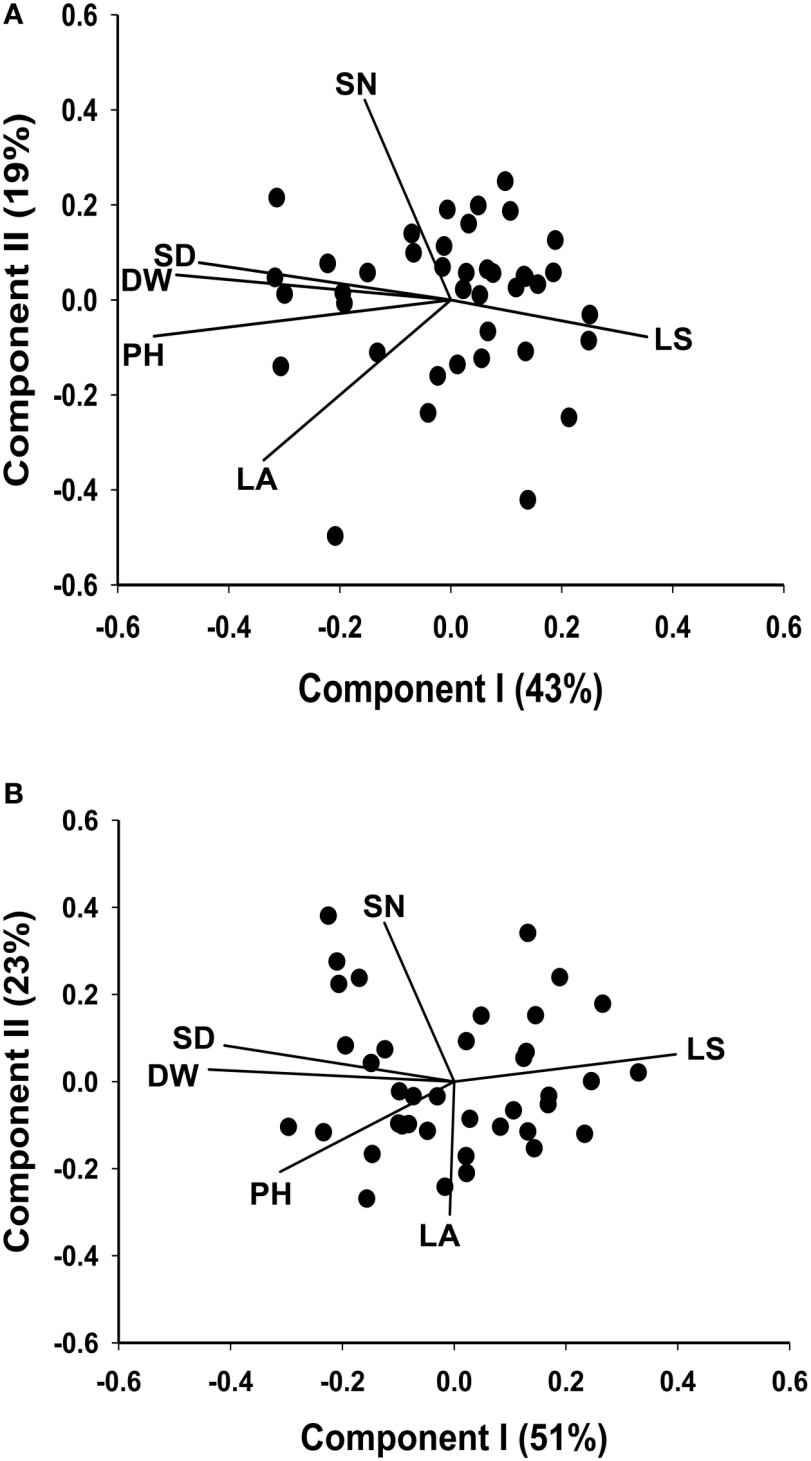
**Biplots based on standardized Best Linear Unbiased Predictor values for seven morphological traits, measured from the 40 half sib families of ***M. officinalis***, evaluated at Yuzhong (A) and Linze (B)**. In each of the biplots Components I and II account for most of the total variation. The vectors represent the traits: LS, leaf to stem ratio; LA, leaf area; PH, plant height; DW, herbage dry weight; SD, stem diameter; SN, stem number.

The seven plant trait responses at the locations Yuzhong and Linze are presented in the two biplots, (Figures 3A,B). In Figure 3A, based on breeding line performance at Yuzhong, the first and second principal components accounted for 43 and 19% of the total variation, respectively. Based on the line performance at Linze, the first principle component explained 51% of the total trait variation, and the second principle component explained 23% (Figure 3B).

There were differences in trait association across the two locations Yuzhong and Linze. The traits DW, SD, PH, SN, and LA showed a strong positive correlation at Yuzhong (angles between the directional vectors are at < 45°). At Linze, DW was positively correlated with SD and PH similar to that showed in Yuzhong. However, SN and LA showed a weak positive associated with DW (Figures 3A,B).

### Cluster analysis

Clustering of the 40 HS families, together with the 6 parental germplasm accessions and 2 check cultivars, was truncated at the four group level. Group 4, the largest group contained 17 members, followed by group 1, group 3 and group 2, which contained 14, 11, and 6 members, respectively (Table 4). As indicated by the Figure 2, the check cultivars were both in group 1. The parental germplasm accessions P1, P2, P3 and P4, P5, P6 were in groups 3 and 1, respectively. The trait means for each group (Table 4) indicated that the members in group 3 had high DW and low coumarin content, and those in group 1 had low coumarin content and intermediate expression for traits DW, PH, SD, and SN. The members in group 4 showed characteristics of a small plant type with high coumarin content. The highest expression for coumarin was in group 2. Groups 3 and 1 had higher SY expression in comparison to groups 4 and 2.

**Table 4:** **Trait means for each of the 4 half sib family groups generated from pattern analysis**.

**Group**	**No. members**	**DW**	**LS**	**PH**	**SD**	**SN**	**LA**	**SV**	**Cou**	**SY**
1	14	104	0.995	162	1.45	7.50	7.74	2.52	0.365	6.07
2	6	123	0.849	164	1.80	7.75	7.58	2.41	1.986	1.99
3	11	157	0.905	168	2.00	8.73	7.87	1.65	0.405	6.54
4	17	73	1.125	150	1.20	7.49	7.58	3.47	1.561	3.00

*LS, leaf to stem ratio; SR, spring vigor; LA, leaf area (cm^2^); PH, plant height (cm); DW, herbage dry weight (g/plant); SD, stem diameter (cm); SN, stem number; Cou, coumarin (% of dry matter); SY, seed yield (g/plant)*.

### Phenotypic and genotypic correlation

A range of genotypic and phenotypic correlation coefficients are presented in Table 5. These coefficients range from strong to weak positive and negative pairwise associations among the 7 traits. Of the special interest are the phenotypic and genotypic correlations between DW and the other traits. There was strong positive phenotypic correlation between DW and the traits SD, PH and SN, and strong negative phenotypic correlation with LS and SV. These results are further supported by the directional vectors in the biplots (Figures 2, 3A,B). In comparison to phenotypic correlation, the estimated genotypic correlation coefficients for all 7 traits showed similar types of pairwise association (Table 5).

**Table 5:** **Genotypic (***r***_***g***_) (lower triangle) and phenotypic (***r***_***P***_) (upper triangle) correlation coefficients, between traits base on the 40 ***M. officinalis*** half sib families, the six parental germplasm accessions and the two check cultivars, evaluated across two locations, Yuzhong and Linze**.

	**DW**	**LA**	**L_S**	**PH**	**SD**	**SN**	**SV**
DW		0.086	−0.371^**^	0.477^**^	0.658^**^	0.336^**^	−0.835^**^
LA	0.021		−0.023	0.116	0.166^*^	−0.070	−0.121
L_S	−0.129	−0.003		−0.227	−0.303	−0.037	0.330^**^
PH	0.082	0.037	−0.131		0.130^*^	0.127^*^	−0.510^**^
SD	0.051	0.011	−0.042	0.029		0.264^**^	−0.528^**^
SN	0.116	−0.045	−0.047	0.019	0.035		−0.361^**^
SR	−0.187	−0.029	0.139	−0.119	−0.057	−0.129	

*LS, leaf to stem ratio; SV, spring vigor; LA, leaf area (cm^2^); PH, plant height (cm); DW, herbage dry weight (g/plant); SD, stem diameter (cm); SN, stem number*.

*, ** *Significant at p < 0.05 and p < 0.01*.

## Discussion

Previous studies on genotypic variation within *Melilotus* spp. have mainly focused on interspecific comparisons for traits such as coumarin content (Nair et al., 2010), salinity, waterlogging tolerance (Rogers et al., 2008), and also on phylogenic relationships (Di et al., 2015) and genetic diversity (Di et al., 2014; Wu et al., 2016). The significant (*P* < 0.05) genotypic variation and high to moderate line mean repeatability reported from our study, indicates the potential for genetic improvement of the nine traits examined. There are no reported studies in *M. officinalis* similar to ours that estimate the magnitude of genotypic variation for key traits such as DW, Cou, PH, and SY.

Phenotypic variation, expressed as ranges, has been reported for some morphological traits. Klebesadel (1992) reported 2 year means of PH of *M. officinalis* ecotypes ranging from 112 to 145 cm. Second year mean plant height (PH) measured in our study ranged from 144 to 188 cm. Martino et al. (2006) reported a range of coumarin content between 0.12 and 0.39% based on different extraction methods. Nair et al. (2010) reported coumarin content measured from 27 *M. officinalis* accessions ranging from 0.09 to 0.61% of dry matter. Our study indicated a coumarin content that ranged from 0.04 to 0.91% of dry matter. Herbage dry matter from single plants has been reported from experiments conducted under glasshouse conditions (Rogers et al., 2008). There is a lack of information on morphological traits measured under field conditions. Results from our study on the genotypic variation for the traits LS, SD, SN, LA, SV and SY, measured under field conditions, will be valuable to *Melilotus* breeders. Information on the magnitude and significance of the genotypic and environmental components of phenotypic variation for important traits will provide a basis for the development of efficient breeding methods for their improvement (Moll and Stuber, 1974). Results from the present study showed that there was significant genotypic variation among the 40 HS families at each location, Yuzhong and Linz, and also across these two locations for all the traits measured. High genotypic variation was present for DW, SV, and SD at Yuzhong and LA, SD, DW, and SV at Linze. These results, together with the relatively high HS family mean repeatabilities estimated, indicate the potential genetic variation available, within the new *M. officinalis* breeding population, for improvement of these traits through selection and breeding.

Forage plants are utilized across a wide range of environments, which include different climates, soil types and grazing systems (Breese, 1969). The presence of genotype-by-environment interactions complicates selection of material for broad adaptation due to unreliable performance across environments (Comstock and Moll, 1963; Cooper and Byth, 1996). Quantifying the magnitude and understanding the causes of genotype-by-environment interaction can be helpful when planning breeding strategies (Milligan et al., 1990; Basford and Cooper, 1998). Caradus (1993) reported that a range of traits in white clover, especially yield-related traits, were sensitive to genotype-by-environment interactions. A similar result in white clover was reported by Jahufer et al. (1999). In our study, the genotype-by-environment interactions were significant for most traits except for the traits LS and LA. This indicates the importance of multi-site evaluation in *M. officinalis* breeding programs when focusing on broad adaptation. The application of multisite testing in breeding programs to investigate the effect of genotype-by-environment interaction on line performance has been reported for forage grass and legume species such as perennial ryegrass (Easton et al., 2015), switchgrass (Jahufer and Casler, 2015), alfalfa (Hill and Baylor, 1983), and white clover (Ballizany et al., 2012).

The association among the traits measured in our study was examined using a combination of phenotypic and genotypic correlation with pattern analysis. The estimates of phenotypic and genotypic correlation coefficients supported the association among traits indicated in the biplots. The positive and significant phenotypic association of DW with traits PH, SD, and SN, predicts a positive correlated response in all these traits when any one of them is selected for individual. This relationship will be useful in a breeding program. The strong positive correlation between DW and SY shown in the biplot (Figure 2) indicates that selection for herbage yield would also result in increasing seed yield. Significant correlation of forage yield and seed yield was also demonstrated in other legumes (Iannucci and Martiniello, 1998; Guler et al., 2001; Cakmakci et al., 2006). Our study indicated negative phenotypic and genotypic correlation between DW and LS. The LS is used as an indicator of digestibility and intake in forage (Kephart et al., 1990). This result implies a trade-off between herbage yield and quality. Julier et al. (2000) also estimated significant negative correlation between DW and LS in alfalfa, which is similar to *M. officinalis* in vegetative form (Whitson et al., 1992).

The strong negative relationship between SV and DW suggests that measurement of spring vigor, at a very early stage of plant growth, could serve as an indirect selection criterion for increasing herbage yield for *M. offcinalis* grown in western China (Table 5). This will increase the efficiency of current breeding methods, especially when dealing with the biennial forage specie like *M. officinalis*. Similar results were reported from studies on common vetch (Cakmakci et al., 2006). The negative phenotypic correlation between the traits DW and Cou shown in our study (Figure 2) indicates the possibility of identifying HS families with a combination of high herbage dry weight and low coumarin content expression. This association will be of significant importance in our *M. officinalis* breeding program. Hofmann and Jahufer (2011) showed negative association between flavonoid accumulation and biomass using multivariate analysis.

Pattern analysis has been successfully used to summarize complex genotype-by-environment (Cooper et al., 1993a; Zhang et al., 2006) and genotype-by-trait (Jahufer et al., 1999; Davodi et al., 2011) data matrices. Jahufer et al. (1999) successfully identified superior white clover full-sib families based on seven morphological traits using a combination of principle component and cluster analysis. Davodi et al. (2011) used pattern analysis to summarize the performance of 200 alfalfa germplasm accessions, based on 12 traits, for use in the improvement of yield and quality. In our study, pattern analysis generated four groups (Figure 3), where group 3 consisted of HS families with above average performance for DW and below average performance for Cou. Group 3 consisted of 11 members, which included the parental germplasm accessions P1, P2, and P3. All the HS families in group 3 had a higher expression of the traits DW, SD, and SY in comparison to both commercial checks. The breeding lines in group 3 will be polycrossed to produce a breeding population that will be used in the recurrent selection program to develop new cultivars of *M. officinalis* with high herbage yield and low coumarin content for the Loess Plateau region in China.

## Conclusion

The estimates of genotypic variation and HS family mean repeatability indicate the potential genetic variation available for all the traits examined in our study. These estimates also indicate the potential to develop cultivars with increased forage yield and low coumarin content. The significant genotype-by-environment interaction estimated for the traits DW, PH, SD, SN, and SV across the two environments, Yuzhong and Linze, indicate the importance of multi-environment evaluation trials in our *M. officinalis* breeding program. The breeding population developed by polycrossing the HS families within group 3, identified using pattern analysis, will provide a significant breeding pool for *M. officinalis* cultivar development in China.

## Author contributions

KL, MJ, JZ, and YW conceived the topic. KL, FW, HD, and XM performed the experiments. KL and MJ analyzed all statistical data. KL wrote the manuscript. All authors revised the manuscript. We thank National Plant Germplasm System (NPGS) for offering the *Melilotus officinalis* seeds.

### Conflict of interest statement

The authors declare that the research was conducted in the absence of any commercial or financial relationships that could be construed as a potential conflict of interest.
